# MIXED METHODS OPTIMIZATION OF BEHAVIORAL SLEEP TREATMENT FOR GRANDPARENTS RAISING GRANDCHILDREN

**DOI:** 10.1093/geroni/igae098.0134

**Published:** 2024-12-31

**Authors:** Melanie Stearns, Ashley Curtis, Christina McCrae

**Affiliations:** University of South Florida, Tampa, Florida, United States; University of South Florida, Tampa, Florida, United States; University of South Florida, Tampa, Florida, United States

## Abstract

Grandparents raising grandchildren (GRG) report greater sleep problems compared to non-caregiving grandparents. Cognitive Behavioral Therapy for Insomnia (CBT-I) is an evidence-based treatment for insomnia; however, the needs of older individuals caring for young children differ from the typical adult who receives CBT-I. Thus, we examined behavioral sleep treatment preferences and collected qualitative and quantitative sleep data from GRG to inform the adaptation of CBT-I for them. The sample consisted of 99 grandparents (Mage=53.62, 70% female) who reported being the primary caregiver of their grandchild (>17 years old). Participants reported on treatment preferences and provided open-ended responses to what negatively impacts their sleep and what they think might improve their sleep. Participants also completed the Pittsburg Sleep Quality Index (PSQI). Based on SOL (Mean=43min) reported on the PSQI, 55% (N=56) of GRG met insomnia criteria. Most GRG (54%) preferred online treatment (27%-in person, 18%-telehealth) and 60% reported they would rather have a behavioral treatment instead of sleep medication to improve their sleep. GRG reported that their stress, anxiety, family issues, and poor grandchild sleep negatively impact their sleep and that stress reduction, greater social support, bedtime routines, and better sleep habits for their grandchildren would likely improve their sleep. These results suggest that GRG prefer online behavioral sleep treatment compared to other modalities (in-person, telehealth) and sleep medication. They may also benefit from a CBT-I which also targets grandchild sleep, reduces stress/anxiety, and helps promote more positive family relationships. Thus, there is a need to adapt CBT-I for their needs.

